# Salt–Drought Co-Stress Impairs Root Ultrastructure, Remodels Rhizosphere Bacteria, and Suppresses Peanut (*Arachis hypogaea* L.) Yield in Saline-Alkali Soil

**DOI:** 10.3390/plants15142116

**Published:** 2026-07-09

**Authors:** Yang Xu, Chen Zhang, Zipeng Yu, Qing Guo, Feifei Qin, Ming Li, Zhimeng Zhang, Hong Ding

**Affiliations:** 1 Shandong Peanut Research Institute, Shandong Academy of Agricultural Sciences, Qingdao 266100, China; xy52120092661@163.com (Y.X.); jone007@126.com (Q.G.); jialing_300@163.com (F.Q.); 2 College of Advanced Agricultural Sciences, Zhejiang Agriculture and Forestry University, Hangzhou 311300, China; 2023201011004@stu.zafu.edu.cn; 3 The Key Laboratory of Plant Development and Environmental Adaptation Biology, Ministry of Education, School of Life Sciences, Shandong University, Qingdao 266237, China; yzp52120090916@163.com; 4 College of Advanced Agriculture and Life Sciences, Weifang University, Weifang 261061, China; lm11a@163.com

**Keywords:** salt and drought combined stress, cell ultrastructure, rhizobacterial community, soil metabolite, peanut yield, saline-alkali area

## Abstract

Background: Peanuts (*Arachis hypogaea* L.) cultivated in saline-alkali areas frequently endure drought stress. Yet, mechanistic research on combined salt–drought stress for peanut growth and yield remains scarce. Methods: A pot culture experiment was conducted to investigate the impacts of short-term drought imposed at the flowering stage on peanuts grown in saline-alkali soil. We comprehensively assessed peanut agronomic traits, cell ultrastructure, physicochemical properties, hormone change, rhizobacterial community, and rhizosphere soil metabolic profiles between single salt stress and salt and drought co-stress. Results: Our study reveals that co-stress markedly suppressed peanut yield, with 100-pod weight, 100-seed weight, pods per plant, and pod yield per plant reduced by 3.16%, 12.79%, 16.65%, and 22.14% relative to salt-only treatment. Combined stress triggered more severe ultrastructural alterations, cell wall degradation, and tissue deformation. It also degraded soil quality by lowering available phosphorus, alkaline hydrolyzable nitrogen, and available potassium. Meanwhile, co-stress reduced the relative abundance of nitrogen-cycling and plant growth-promoting rhizobacteria, alongside depleted beneficial sugar metabolites in rhizosphere soil. These shifts jointly constrain peanut growth and productivity. Conclusions: Thus, it is imperative to implement timely irrigation practices to avoid drought during peanut cultivation in saline-alkali areas, particularly during the flowering stage.

## 1. Introduction

Peanut (*Arachis hypogaea* L.) is a globally important oil and cash crop, with seeds rich in oil (36–54%), protein (16–36%), and carbohydrates (10–20%) [1]. Owing to the limited arable land and requirements for developing regional agriculture, peanut cultivation has been experimentally extended to saline-alkali soils in northern China, particularly in coastal zones such as the Yellow River Delta. Local saline-alkali soils feature high pH and soluble salt loads dominated by NaHCO_3_ and Na_2_CO_3_ [2]. Coupled with low rainfall and insufficient freshwater supplies in this zone, peanuts frequently suffer simultaneous salt and drought stress throughout their growth cycle [3].

Salt and drought jointly disrupt plant growth, reproduction, and fertility by triggering shared physiological disturbances such as osmotic imbalance, cellular dehydration, and reactive oxygen species (ROS) overproduction [4]. Chloroplasts, mitochondria, peroxisomes, and apoplast represent the primary organelle-derived sources of ROS under stress, where disordered photosynthetic electron transport, disrupted respiratory chains, and enhanced photorespiration jointly drive ROS burst [5]. Excess ROS act as cytotoxic substances that induce lipid peroxidation, metabolic imbalance, and irreversible damage to organelle ultrastructure, while basal levels of ROS function as vital secondary signal carriers to relay environmental stress signals to intracellular regulatory pathways [6]. Under co-occurring salt and drought stress, ROS signaling forms a bidirectional feedback loop with abscisic acid (ABA): ROS activate ABA biosynthesis, and accumulated ABA further boosts NADPH oxidase-dependent ROS production [7]. Moreover, ABA induces the transcription of genes related to the ascorbate-glutathione cycle in the ROS scavenging system to avoid excessive accumulation of ROS. This tight ROS–ABA crosstalk governs plant stress adaptation and growth [7]. Dual stress also suppresses photosynthetic capacity, transpiration rates, and root functionality, ultimately reducing crop yield [8]. Salinity additionally causes toxic Na^+^ and Cl^−^ ion accumulation to worsen physiological damage [4].

Over millions of years of evolution, plants have developed remarkable plasticity and sophisticated mechanisms to sense and mitigate the Na^+^ imbalance and hyperosmotic stress caused by salt and drought stress [9]. Core regulatory modules, including the salt overly sensitive (SOS) pathway and MAP kinase cascades, are rapidly activated upon stress exposure [10,11,12]. Multiple phytohormones (ABA, auxin, gibberellin (GA), salicylic acid (SA), and jasmonic acid (JA)) coordinate complex signaling networks to orchestrate plant stress responses [9]. Plants also synthesize osmolytes, antioxidants, and molecular chaperones to scavenge toxic ROS and strengthen osmotic tolerance [13,14].

Beyond intrinsic plant physiological adjustments, rhizosphere bacterial communities and soil metabolites exuded by roots are critical external regulators of plant performance under abiotic stress [15,16]. Certain root exudates recruit plant growth-promoting rhizobacteria (PGPR) to boost stress resistance [17], while others attract pathogen-dominated “diseased” microbiome to negatively impact the rhizosphere microbial community structure and plant stress resistance [18]. Moreover, rhizobacterial community composition can also be a key driver of metabolic shifts in rhizosphere soil [19]. To date, few studies have systematically characterized functionally beneficial rhizobacteria and protective soil metabolites under combined salt–drought stress. More importantly, it remains unclear how superimposed short-term flowering-stage drought differs from single salt stress in altering peanut physiology, rhizosphere microecology, and final pod yield, constituting a critical knowledge gap limiting targeted cultivation management for saline-alkali peanut fields.

To fill this research gap, this study had two clear objectives: (1) to compare differences in peanut pod yield, root and pod cell ultrastructure, rhizosphere soil physicochemical properties, rhizobacterial community composition, and soil metabolite profiles between single salt stress and combined salt and short-term flowering drought stress; (2) to reveal the integrated regulatory mechanisms linking plant hormone homeostasis, root ultrastructural integrity, and peanut yield under co-stress. We proposed the core hypothesis that short-term flowering drought superimposed on saline-alkali conditions will aggravate yield loss via altering root ultrastructural structure, hormone contents, rhizosphere soil bacterial taxa, or metabolite profiles. We adopted an integrated multi-indicator approach combining agronomic traits, hormone detection, cell ultrastructure, untargeted rhizosphere metabolomics, rhizobacterial community, and soil physicochemical properties to verify this hypothesis. The findings provide theoretical support for stable peanut production and sustainable exploitation of coastal saline-alkali land.

## 2. Results

### 2.1. Short-Term Drought Exacerbates the Decline in Pod Yield of Peanuts in Saline-Alkali Areas

Notably, in saline-alkali areas, peanut morphology and pod yield exhibited heightened sensitivity to combined salt and drought stress as opposed to the impact of salt stress alone (Table 1). In 2024, a short drought imposed at the flowering stage (SD) significantly suppressed peanut morphology growth compared to single salt stress (S) under saline conditions. Main stem height dropped by 11.00%, and lateral branch length decreased by 13.79% in SD relative to S (*p* < 0.05). Multiple yield components also declined markedly under co-stress (Table 1). Compared with the S group, SD reduced 100-pod weight by 3.16%, 100-seed weight by 12.79%, pod number per plant by 16.65%, and single-plant pod yield by 22.14% (all *p* < 0.05). Collectively, co-stress lowered peanut yield via reducing the number of pods per plant, the weight of both 100 seeds and 100 pods, and the overall pod yield per plant. Crucially, the flowering stage is a water-sensitive critical period for peanut. Even a short drought occurring at this stage aggravates final yield loss on saline-alkali land.

### 2.2. Effects of Short-Term Drought on Cell Ultrastructure in Saline-Alkali Areas

**Figure 1:**
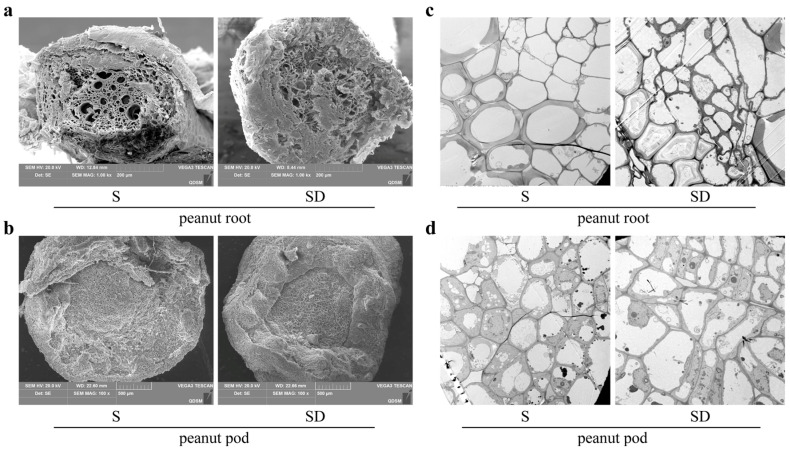
Scanning electron microscopy (SEM) and transmission electron microscopy (TEM) micrographs of peanut roots and pods under single salt stress (S) and combined salt and drought stress (SD). All ultrastructural comparisons are restricted to the S and SD groups. SEM images showing morphological variations in specimens prepared from the peanut root (**a**) and pod (**b**) under the two stress groups. SEM images were acquired at a magnification of 1.00 k ×, with a scale bar of 200 μm for image (**a**) and 500 μm for image (**b**). TEM images showing intracellular ultrastructural features of peanut root (**c**) and pod (**d**) under S and SD. Scale bars = 20 µm in images (**c**,**d**). Treatment definitions: S, peanuts grown in saline-alkali soils; SD, peanuts cultivated in saline-alkali soils with short-term drought stress imposed at the flowering stage.

Scanning electron microscopy (SEM) images provided insights into the surface micromorphology of the samples, while transmission electron microscopy (TEM) images unveiled intracellular components. Microscopic examination of peanut roots revealed that the vascular bundles in both the S group and SD group were disorganized and deformed, with the central medullary cavity cracked (Figure 1a). The extent of damage was more pronounced in the SD group than in the S group (Figure 1a). In contrast, the micromorphology of peanut pods in the SD group and S group were similar, with no obvious differences observed (Figure 1b). TEM images further demonstrated that intracellular micromorphology arrangements became less compact and even deformed in the roots and pods of the SD group compared to the S group (Figure 1c,d). Collectively, combined stresses altered the ultrastructure, disrupted the cell wall, and caused more severe tissue deformation in peanut roots and pods than in the S group, potentially exacerbating the damage inflicted by single salt stress alone.

### 2.3. Synergistic Effects of Combined Drought and Salt Stress on Rhizosphere Soil Physicochemical Properties and Peanut Hormone Contents

**Figure 2:**
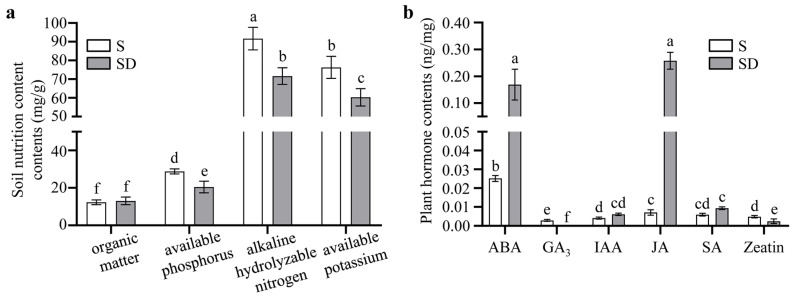
Rhizosphere soil physicochemical properties and different plant hormone contents in peanut roots. (**a**) Rhizosphere soil physicochemical properties assays. (**b**) Different plant hormone contents in peanut roots of different groups. Error bars indicate the SE (*n* = 3). One-way ANOVA Duncan’s test. Different lowercase letters above the columns represent different levels of significance.

Regarding the rhizosphere soil properties, combined salt and drought stress led to a notable decrease in the contents of soil available phosphorus, alkaline hydrolyzable nitrogen, and available potassium, with reductions of 29.10%, 21.87%, and 20.98%, respectively (*p* < 0.05, Figure 2a). Conversely, the contents of organic matter remained basically unchanged between the SD group and S group (Figure 2a). Depressed soil nutrient supply under co-stress limits peanut growth and reduces pod yield.

To determine how peanut roots respond to the inferior soil quality under SD stresses in terms of hormone levels, we quantified various phytohormones—including auxin (IAA), gibberellic acid (GA_3_), ABA, JA, zeatin, and SA within peanut roots (Figure 2b). Our findings revealed a significant increase in ABA and JA levels in the SD group when compared to those in the S group, whereas the increments in IAA and SA levels were relatively minor, and the differences observed did not reach statistical significance within the SD group roots (*p* < 0.05; Figure 2b). Conversely, GA_3_ and zeatin levels decreased in the roots of the SD group under the combined treatment (*p* < 0.05; Figure 2b). Notably, with the exception of zeatin and GA_3_, most plant hormones exhibited an elevation in peanut roots of the SD group, indicating amplified stress response under simultaneous salt and drought stress [9].

### 2.4. Metagenomic Sequencing Analysis of the Bacterial Community Diversities of Different Rhizosphere Soils

Metagenomic sequencing was employed to investigate the effects of the combined salt and drought stress on the composition and diversity of rhizosphere soil bacterial communities. Alpha diversity indices, which serve as indicators of microbial ecosystem richness and diversity, revealed that both peanut rhizosphere soil samples exhibited comparable and elevated levels of species richness and diversity (Table 2).

**Figure 3:**
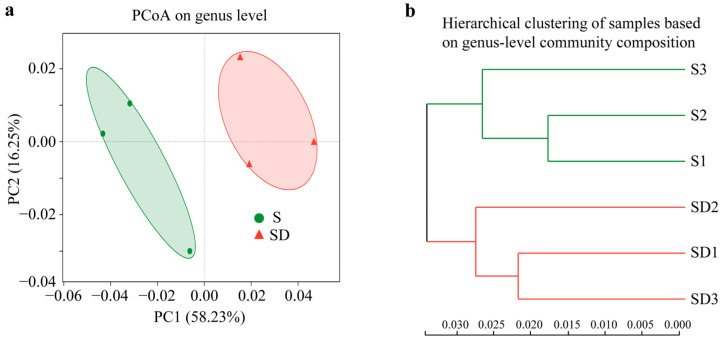
Beta diversity analysis. (**a**) Principal coordinate analysis (PCoA). In this PCoA plot, points sharing the same color belong to the same soil group, and these groups are further delineated by ellipses for clarity. Green circles represent samples from group S, while red triangles represent samples from group SD. (**b**) Hierarchical clustering analysis is employed to illustrate the clustering of soil groups according to their similarity. Green branches correspond to all replicates of group S (S1, S2, S3), and red branches correspond to all replicates of group SD (SD1, SD2, SD3).

Furthermore, we conducted a beta diversity analysis to assess the overall shifts in rhizobacterial community composition between comparable groups. Principal coordinate analysis (PCoA) unveiled a significant variance between soil groups, with the S group and SD group displaying distinct rhizobacterial community compositions that were clearly separated along the coordinate axis (Figure 3a). In hierarchical clustering analysis, the branch length between soil groups indicated the degree of similarity between them, revealing that rhizobacterial community structures varied significantly across different soil groups and were widely dispersed. Conversely, duplicate samples within the same groups exhibited similarity and clustered closely together (Figure 3b). These results suggest that drought stress during the flowering stage reshapes rhizobacterial assembly compared to the effects of salt stress alone in saline-alkali areas.

### 2.5. SD Stresses Reshaped the Rhizobacterial Community

**Figure 4:**
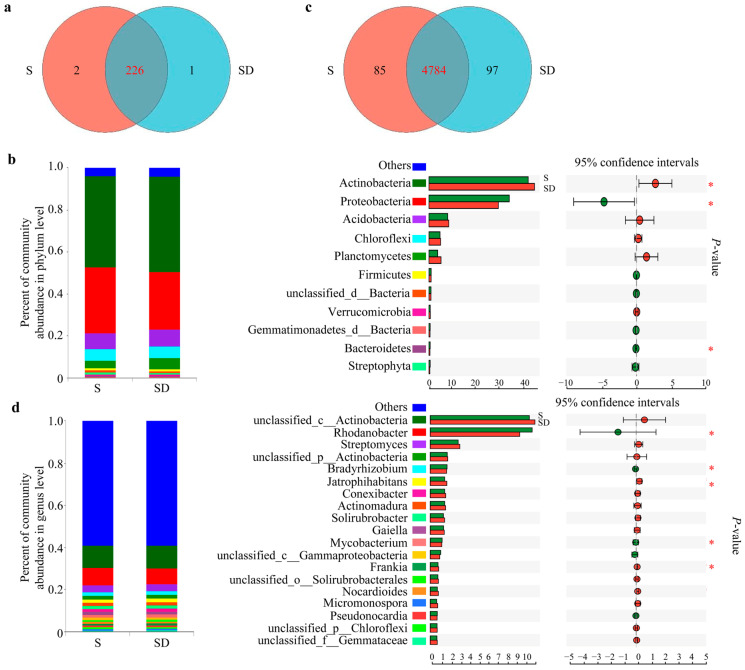
Combined salt and drought stress altered the peanut rhizobacterial community structure. (**a**) Venn diagram showing total and shared phyla of rhizobacteria in the two distinct treatments. The red circle represents group S and the blue circle represents group SD, overlapping gray area denotes shared phyla between two groups. (**b**) Phylum-level taxonomic composition in peanut rhizosphere soils from three duplicates. Left: relative abundance of each phylum; Right: Wilcoxon rank-sum test illustrating abundance differences with 95% confidence intervals. Green horizontal bars and dots stand for group S, and red horizontal bars and dots stand for group SD. *, *p* < 0.05. (**c**) Venn diagram displaying total and shared rhizobacterial genera between the two groups. The red circle corresponds to group S and the blue circle corresponds to group SD, overlapping gray region represents shared genera of the two treatments. (**d**) Genus-level taxonomic composition averaged from three replicates. Left: relative abundance of each genus; Right: Wilcoxon rank-sum test showing intergroup abundance differences with 95% confidence intervals. Green horizontal bars and dots stand for group S, and red horizontal bars and dots stand for group SD. *, *p* < 0.05. “Unclassified” indicates genera without taxonomic annotation that were directly obtained from the database through sequence alignment.

To explore the influence of SD stresses on the diversity of rhizobacterial communities, we conducted a detailed analysis of the rhizobacterial composition in each soil sample, focusing on both phylum and genus classifications (Figure 4). Venn diagrams indicated a notable similarity in the distribution of bacterial phyla between the two soil groups (Figure 4a). Specifically, the SD group and S group had only one and two unique phyla, respectively, with a substantial overlap of approximately 98.69% (Figure 4a). Key phyla such as Actinobacteria, Proteobacteria, Acidobacteria, Chloroflexi, and Planctomycetes were dominant across all soil samples, constituting more than 85% of the bacterial taxa (Figure 4a). Notably, in the SD group, there was a significant decline in the populations of nitrogen-cycling-related phyla Proteobacteria and Bacteroidetes by 11.60% and 11.15% compared to the S group, respectively, while Actinobacteria saw an increase of 4.40% (Figure 4b). At the genus level, the S group and SD group exhibited 85 and 97 unique genera, respectively, with a shared presence of approximately 96.34% (Figure 4c). The relative abundance of all bacterial genera remained below 10.85%, highlighting that the peanut soils remained a challenging reservoir of biodiversity at this taxonomic level (Figure 4d). Among these, nitrogen-cycling-related and plant growth-promoting genera, *Rhodanobacter*, *Bradyrhizobium*, and *Mycobacterium* showed a marked decrease in abundance within the SD group compared to the S group, with the sole exception of *Jatrophihabitans*, which demonstrated a significant increase (Figure 4d). Further validation via Linear Discriminant Analysis (LDA) Effect Size (LEfSe) analysis corroborated the significant decrease in rhizobacteria in the SD group (Figure S1). Combined salt and drought stress suppresses beneficial rhizobacterial populations linked to nitrogen cycling and plant growth promotion, potentially posing a threat to peanut growth and its ability to tolerate stress.

### 2.6. SD Stresses Altered Rhizosphere Soil Metabolic Profiles

**Figure 5:**
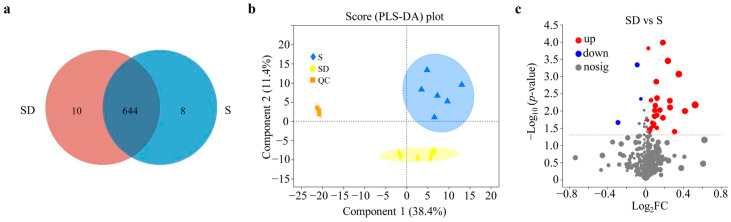
Variation in soil metabolic profiles under salt stress and combined stress. (**a**) Venn analysis of the soil metabolites unique or co-existing in the two rhizosphere soil groups. The red circle represents group SD, the blue circle represents group S, and the gray overlapping region indicates shared metabolites between the two treatments.(**b**) Partial least-squares-discriminant analysis (PLS-DA) is utilized to discriminate between the soil metabolic profiles of the different treatment groups. Blue diamond markers and light blue shaded ellipse denote samples from group S; yellow square markers and pale yellow shaded ellipse represent group SD samples; orange square markers with light orange shadow correspond to quality control (QC) samples. (**c**) Volcano plot of the data of differential metabolites under salt stress and combined stress. Up: red dots represent significantly upregulated differential metabolites in combined stress-treated groups compared to single salt-treated groups. Down: blue dots represent significantly downregulated differential metabolites in combined stress-treated groups compared to single salt-treated groups. Gray dots indicate metabolites with non-significant expression changes (nosig). The horizontal red dotted line marks the significance threshold of *p* = 0.05 for screening differential metabolites.

Utilizing nontargeted metabolomics analysis based on ultra-high-performance liquid chromatography-tandem mass spectrometry (UHPLC-MS/MS), we successfully identified 645 metabolites in positive ion mode and 176 in negative ion mode within the soil samples (Table S1). Among them, a total of 239 in positive ion mode and 44 in negative ion mode were specifically identified and named by referencing Kyoto Encyclopedia of Genes and Genomes (KEGG) pathways (Table S1). The Venn diagram demonstrated 10 and 14 unique metabolites with substantial differences in the S group and SD group, respectively, and 811 differential metabolites coexisted in both soil groups (Figure 5a). Partial least-squares discrimination analysis (PLS-DA) demonstrated a distinct separation between S and SD groups, indicating that combined stress changed the soil metabolic profiles (Figure 5b). The close clustering of quality control (QC) samples in both positive and negative ion modes indicated the experiment’s reproducibility and the stability and reliability of the test data (Figure 5b). The volcanic plot of metabolites given the positive and negative ion modes is shown in Figure 5c. There were 40 (including 32 upregulated and eight downregulated) metabolites that exhibited differential accumulation in the SD group vs. the S group according to a certified VIP score (VIP > 1) and *p* < 0.05 criteria.

Utilizing KEGG pathway predictive analysis, we successfully mapped 11 out of the 40 identified differentially accumulated metabolites (DAMs) to their corresponding KEGG pathways. Notably, a substantial fraction of these DAMs was associated with the biosynthesis of secondary metabolites, amino acid metabolism, and carbohydrate metabolism (Table 3, Figure S2). Several sugar metabolites, including sucrose and maltotriose, were notably (*p* < 0.05) decreased in the SD rhizosphere soils compared to single S soils, whereas the levels of styrene, pelargonidin, and actinidine increased markedly in these soils (*p* < 0.05, Table 3). Among them, pelargonidin serves as a pivotal crosstalk metabolite, showing pronounced upregulation that coordinates flavonoid biosynthesis, anthocyanin biosynthesis, and the broader biosynthesis of secondary metabolites (Table 3). Conversely, sucrose, another crosstalk metabolite, exhibited a significant decline, primarily participating in galactose metabolism, starch and sucrose metabolism, biosynthesis of secondary metabolites, ABC transporters, and the phosphotransferase system (Table 3). Overall, these findings reinforce the notion that SD stresses trigger changes in rhizosphere soil metabolic profiles.

### 2.7. Correlation Analysis Between Rhizobacterial Communities and Soil Metabolites

**Figure 6:**
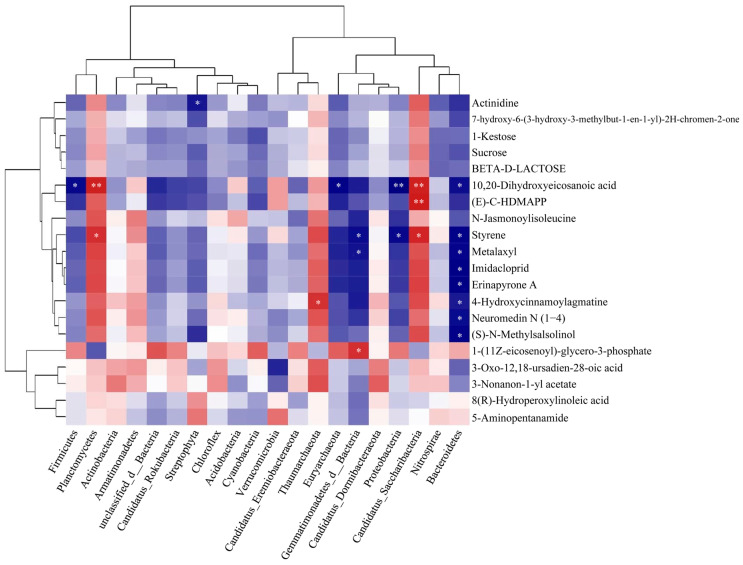
Correlation analysis between rhizobacteria and soil metabolites. Red boxes represent positive correlations, while blue boxes represent negative correlations (Pearson’s correlation, *n* = 6, *p* < 0.05). White asterisks indicate statistical significance: *, *p* < 0.05; **, *p* < 0.01. Red denotes positive correlations between bacterial phyla and metabolites, and blue represents negative correlations.

Through Pearson’s correlation analysis (*p* < 0.05; Figure 6), we explored the correlations between rhizosphere soil bacteria and metabolites that exhibited significant differences across various treatments, identifying associations between 20 metabolites and 20 bacterial phyla. Proteobacteria showed significant negative correlations with 10,20-dihydroxyeicosanoic acid and styrene. Candidatus_Saccharibacteria had strong positive correlations with 10,20-dihydroxyeicosanoic acid, (E)-C-HDMAPP and styrene (Figure 6). Additionally, our analysis revealed that the relative abundances of 10,20-Dihydroxyeicosanoic acid were significantly positively correlated with Candidatus_Saccharibacteria and Planctomycetes, but negatively correlated with Firmicutes, Euryarchaeota, and Bacteroidetes (Figure 6). Streptophyta exhibited a strong negative correlation with actinidine, while Thaumarchaeota showed a significantly negative correlation with 4-Hydroxycinnamoylagmatine (Figure 6). Bacteroidetes exhibited a negative correlation with the highest number of metabolites, totaling eight (Figure 6). The observed positive co-occurrences suggest that certain soil metabolites might be secreted by microbial communities, whereas negative co-occurrences could be attributed to specific microbial consumption or degradation processes, as previously reported [19]. These data support that bacterial communities within the peanut rhizosphere soil could potentially play a pivotal role in driving alterations in soil metabolic profiles.

## 3. Discussion

In alignment with the findings of the previous study [20], salt and drought are dominant abiotic hazards restricting peanut productivity in saline-alkali land. Short-term drought superimposed on salinity caused larger declines in 100-pod weight, 100-seed weight, pod number per plant, and single-plant pod yield relative to single salt stress (Table 1). This clearly underscores the compounded negative effects of combined salt and drought stresses on peanut productivity. In this study, we employed a combination of soil metabolomics, metagenomic sequencing technologies, cell ultrastructure analysis, hormone contents, and physicochemical indicator testing in a pot experiment to systematically explore the multi-layered mechanisms driving yield loss under flowering-stage drought in saline-alkali soil. It is important to note that the ultrastructure changes, cell wall damage, and tissue deformation were more severe in the SD group (Figure 1), a phenomenon that may be correlated with suppressed pod productivity (Table 1). Meanwhile, SD significantly depleted rhizosphere available nitrogen, phosphorus, and potassium (Figure 2a), reduced the relative abundance of nitrogen-cycling and plant growth-promoting rhizobacteria, and lowered rhizosphere sugar metabolites (Figure 4, Table 3). These declines in beneficial rhizobacteria and soil metabolites, coupled with the deterioration in soil quality, are also likely to have detrimental consequences on peanut growth and pod yield. Future manipulative experiments including strain inoculation and exogenous metabolite supplementation assays are required to obtain direct causal evidence.

Rhizosphere soil represents a complex ecological system, and a wide range of environmental factors have been demonstrated to influence plant rhizobacterial community compositions [21]. In our study, combined salt and drought treatment reshaped the rhizobacterial composition, with functionally significant beneficial phyla and genera showing a dramatic decrease compared to soils subjected solely to salt, including Proteobacteria, Bacteroidetes, *Rhodanobacter*, *Bradyrhizobium*, and *Mycobacterium* (Figure 4b,d). Proteobacteria have earned recognition for their pivotal roles in nitrogen fixation and the decomposition of organic matter, which can supply plants with nutrients in a readily available form [22]. Moreover, Proteobacteria demonstrate plant growth-promoting traits by facilitating nitrogen fixation and enhancing the solubilization of nutrients [23]. Members of Bacteroidetes play a critical role in maintaining plant health and function, thereby creating a conducive microenvironment for plant growth [24,25]. As a genus within the Proteobacteria phylum, *Rhodanobacter* species are chemoautotrophic bacteria that are capable of simultaneously participating in Hg^0^ oxidation, nitrification, and denitrification processes [26]. *Bradyrhizobium* populations are one of the famous plant growth-promoting rhizobacteria, which can play a key role in nitrogen cycling [27]. Most *Mycobacterium* strains alleviate crop abiotic stress and suppress pathogenic microbes [28]. The loss of these beneficial taxa under SD may logically impair rhizosphere nitrogen cycling and plant growth-promoting capacity, compromising plant growth promotion and disease suppression functions that are well documented in the above literature [22,23,24,25,26,27,28], thereby weakening peanut stress tolerance. In contrast, the genus *Jatrophihabitans* significantly accumulated under dual stress, pointing to a non-random community filtering process. The core molecular mechanisms linking co-stress to rhizobacterial remodeling remain unclarified. It is still unclear whether such community shifts are primarily governed by altered root exudate profiles, modified soil pH, inter-taxa disparities in osmotic stress tolerance or other uncharacterized rhizosphere environmental drivers. Characterizing the composition and quantity of root exudates under S versus SD conditions using targeted metabolomics would provide a mechanistic bridge between plant physiological stress responses and the observed microbiome shifts.

Several sugar metabolites, including sucrose and maltotriose, were notably (*p* < 0.05) decreased in the SD rhizosphere soils compared to those in single S soils, whereas the levels of styrene, pelargonidin, and actinidine rose markedly in these soils (Table 3). This differential metabolite enrichment aligns with the hypothesis that combined abiotic stresses trigger more complex metabolic reprogramming in plants than individual stressors [29]. Soluble sugars, including sucrose and maltotriose, act as two core functional compounds in rhizosphere ecosystems: they serve as primary carbon energy for plant root growth, and function as chemotactic signaling molecules to recruit beneficial PGPR and sustain nitrogen-fixing microbial metabolic activity [30]. The observed sugar decline under SD may simultaneously disrupt two key rhizosphere processes: it cuts off carbon substrate supply for rhizobacterial nitrogen fixation, and eliminates sugar-derived chemotactic cues that attract *Bradyrhizobium* and *Rhodanobacter*, jointly driving the depletion of beneficial bacterial populations. Despite this clear correlative pattern, dedicated exogenously supplemented sugar trials are needed to confirm this mechanism experimentally. As for elevated secondary metabolites, styrene, pelargonidin, and actinidine are substrates involved in the biosynthesis of secondary metabolites and amino acid-related metabolisms (Table 3). Pelargonidin, a core anthocyanidin substrate, has been reported to help plants resist UV and high salt stress in Buckwheat [31]. Flavonoid and anthocyanin, produced during the biosynthesis of secondary metabolites, can function as ROS scavengers and membrane stabilizers under stress conditions [32,33]. We speculate that elevated rhizosphere pelargonidin and actinidine under combined stress may serve as precursors for flavonoid biosynthesis in peanut roots, and the subsequent flavonoid accumulation could constitute a protective mechanism to counteract osmotic- and ionic stress-triggered oxidative damage. In contrast, the functional role of styrene, a metabolite with reported phytotoxic and antimicrobial activities, remains poorly understood [34]. Moreover, styrene derived from *Bacillus* mycoides acts as a bioactive chemical messenger in soil, which nematodes perceive via AWB olfactory neurons through a conserved G protein–calcium signaling cascade, triggering repellent behavior and even lethal toxicity at elevated concentrations [35]. Distinguishing between protective, signaling, and detrimental roles of styrene represents a critical future direction. It should be emphasized that the current study only observed stress-induced enrichment of these metabolites in rhizosphere soil, without direct functional evidence to validate their biological roles in peanut. Further efforts in functional verification, such as exogenous application of pelargonidin or actinidine, genetic manipulation of their biosynthetic pathways, or styrene toxicology assays, are required in future research to confirm this tentative hypothesis in our experimental material.

The synergistic effects of drought and salt stress induce pronounced ultrastructural changes in peanut cells, culminating in damage to cell walls and plant tissues. Under SD stress, we observed a significant elevation of ABA and JA in peanut roots. ABA activates plasma membrane NADPH oxidase to boost intracellular ROS burst, which amplifies oxidative damage to cell walls and organelles [7]; JA further reinforces ROS signaling and accelerates oxidative damage [36]. Although ABA normally induces stomatal closure to reduce water loss, this response restricts leaf CO_2_ intake and exacerbates photosystem oxidative stress [37]. Unfortunately, leaf photosynthetic indicators were not measured in this pot experiment, so we cannot further link ABA signaling to leaf photodamage. In this study, we speculate that the elevated ABA–JA co-signaling elevates root ROS levels, which accelerates membrane lipid peroxidation and cell wall structural degradation (Figure 1 and Figure 2). At present, we cannot distinguish whether hormone overaccumulation actively drives structural damage or merely passively reflects tissue deterioration under co-stress. Single-cell and spatial transcriptomics, which can resolve stress-responsive transcriptional networks at cellular resolution [38], can be adopted to dissect the root structural damage and spatially differentiated hormone signaling detected in subsequent research to resolve cell-type-specific hormone–ultrastructure regulatory networks.

Soil nutrient status governs the assembly of rhizobacterial communities [39], and altered microbial assemblages reciprocally modify rhizosphere soil physicochemical traits in return [40]. Accordingly, we hypothesize that reduced rhizosphere nutrient availability functions as both a downstream consequence and a reinforcing driver of plant–rhizosphere disturbances under salt–drought co-stress. Flowering-stage drought initially aggravates osmotic and ionic stress in peanut roots; such physiological disruption may remodel rhizobacterial compositions and weaken microbial mineralization activity, thereby reducing bioavailable nitrogen, phosphorus, and potassium in the rhizosphere soil. Nutrient depletion subsequently limits the proliferation of nutrient-dependent beneficial bacteria, further restructuring rhizobacterial communities and suppressing soil nitrogen transformation. Collectively, we propose this bidirectional negative feedback loop accelerates rhizosphere degradation under SD treatments, and targeted functional manipulation experiments are required to verify this hypothesis in follow-up research.

All analyses in our study relied on controlled pot cultivation rather than cultivation in natural saline-alkali field soils, thus failing to mimic layered soil structures, dynamically fluctuating soil moisture, and heterogeneous field microhabitats. In addition, we only imposed short-term drought at the flowering stage, restricting the generalizability of our conclusions to other growth periods or chronic co-stress conditions. Furthermore, the single-time-point sampling design only yields correlative multi-omics and physiological data, meaning all hypothesized regulatory interactions cannot be confirmed without targeted functional manipulation trials. Several core mechanistic uncertainties derived from our correlative dataset await verification via multi-site field experiments in the Yellow River Delta in future studies. Key unresolved questions concern the following: (i) whether reduced rhizosphere sugars restrict carbon availability or disrupt sugar-mediated chemotaxis for PGPR colonization; (ii) the primary driver responsible for reshaping rhizobacterial communities under co-stress; (iii) the dose-dependent dual functions of styrene as a phytotoxin or signal. Addressing these gaps will transform our correlative observations into definitive causal regulatory evidence. Despite these limitations, we propose a correlative mechanistic framework to interpret how combined salt and flowering-stage drought stress suppress peanut pod yield: co-stress induces accumulation of hormone ABA and JA in roots, depletes rhizosphere-available nitrogen, phosphorus, and potassium, reduces the relative abundance of beneficial taxa, and lowers rhizosphere sucrose and maltotriose pools; these concurrent rhizosphere and physiological disturbances collectively trigger severe root ultrastructural deterioration, ultimately constraining peanut pod productivity. Our results provide clear agronomic guidance: timely irrigation at the flowering stage effectively alleviates combined salt–drought stress and mitigates yield losses in saline-alkali peanut cultivation.

## 4. Materials and Methods

### 4.1. Experimental Design and Peanut Planting

Pot experiments were performed in a growth chamber of Shandong Academy of Agricultural Sciences Yellow River Delta Modern Agriculture Research Institute (longitude 118°36′59″, latitude 37°17′30″, Dongying, China), in 2023 and 2024. The peanut cultivar used in this study was cv. Huayu36, generously provided by Prof. Jing Chen from Shandong Peanut Research Institute. Seeds were sown in a transparent acrylic tank (36 cm in diameter and 26 cm tall) with tiny holes in the bottom containing an equal amount (18 kg) of topsoil dug by hand from saline-alkali areas of Shandong Academy of Agricultural Sciences Yellow River Delta Modern Agriculture Research Institute. The soil exhibited the following physiochemical properties: a pH of 8.8, electrical conductivity (ECe) of 5.17 dS m^−1^ [41], organic content of 15.80 g kg^−1^, available nitrogen at 36.98 mg kg^−1^, available potassium at 10.73 mg kg^−1^, and available phosphorus at 284.50 mg kg^−1^. The soils contained approximately 2.50 g kg^−1^ of total soluble salts. Triple compound fertilizer 15–15–15 (N-P_2_O_5_-K_2_O) was applied at a rate of 600 kg ha^−1^ and thoroughly mixed into the soil before peanut planting, on 15 May 2023 and 2 May 2024. Each tank accommodated four full peanut seeds, which were watered every other day to maintain a soil water content of 75% of field capacity, following the methodology outlined [42]. The peanuts were cultivated under optimal growth conditions (16 h light/8 h dark, 300 μmol m^−2^ s^−1^, 28 °C/18 °C (day/night), 60% relative humidity) in a growth chamber [42]. Seeds were sown in the experimental tanks on 19 May 2023 and 6 May 2024, with subsequent harvests on 10 October 2023 and 8 October 2024, respectively.

For the short-term drought treatment groups, the soil water level was reduced to 45% of field capacity for approximately 10 days during the peanut flowering stage [43]. This continued until half of the plants displayed visible signs of drought stress, including leaf curling, wilting, and senescence [43]. Throughout the entire short-term drought treatment period, the growth chamber maintained the same fixed temperature cycle (28 °C/18 °C (day/night)) and a constant relative humidity of 60% to avoid variable transpiration rates and inconsistent drought stress intensity across replicate tanks. This experiment encompassed two distinct treatments: (i) peanuts planted in saline-alkali soils, subjected to single salt stress (denoted as S); and (ii) peanuts grown in saline-alkali soils with the addition of short-term drought stress during the flowering stage, thus experiencing the combined effects of salt and drought stresses (denoted as SD). Each treatment group comprised nine tanks. Subsequently, samples were collected from three tanks 10 days post-stress for measurement of rhizosphere soil physicochemical properties and plant hormones. This sampling time point captured the peak stress response before rehydration. After the drought stress treatment, the remaining six tanks were re-irrigated with water to restore and maintain the soil water content at 75% of field capacity until harvest for electron microscope analysis and pod yield assessments, mirroring the conditions of the S group. The experimental tanks were arranged as a randomized block design.

### 4.2. Rhizosphere Soil Sampling

Rhizosphere soils consisting of soils around the roots and root surface soils were collected at the flowering stage of peanuts, with samples taken under both salt conditions and combined stress scenarios on 8 July 2024, for measurement of soil physicochemical properties and soil metagenomic and nontargeted metabolomic sequencing. To standardize rhizosphere soil sampling, intact peanut root systems were first gently shaken to remove loosely attached bulk soil. Only the tightly adhering soil layer within a unified radial distance of 1–10 mm from root surfaces was then carefully brushed off into sterile sampling bags. Disposable sterile soft brushes were used for each individual plant and applied with consistent brushing strength and angle to eliminate cross-contamination between samples [44,45]. For root surface soil extraction, peanut roots were excised and immediately placed into a centrifuge tube containing 40 mL of PBS buffer (pH 7.0, containing 6.33 g of NaH_2_PO_4_·H_2_O, 16.5 g of Na_2_HPO_4_·7H_2_O, and 200 mL Silwet L-77 per liter). The collected rhizosphere soil samples were promptly frozen in liquid nitrogen to preserve their integrity and subsequently stored at −80 °C until further analysis. All the experiments were performed with 6 biological replicates to ensure the reliability and reproducibility of the results.

### 4.3. Soil Physicochemical Properties Assays

Six biological replicates were set for each treatment group for rhizosphere soil physicochemical properties assays. Soil organic matter content was determined through the external heating technique involving potassium dichromate, followed by the titration method [46]. Soil alkali hydrolyzable nitrogen was extracted utilizing the alkaline hydrolysis diffusion method and subsequently titrated with a standard sulfuric acid solution (5 mmol L^−1^). The determination of soil available phosphorus was carried out via NaHCO_3_ spectrophotometry, with absorbance measured at 880 nm using a UV-759S spectrophotometer from SHIMADZU, Kyoto, Japan. Soil available potassium was quantified employing the ammonium acetate flame photometry technique [47].

### 4.4. Metagenomic Sequencing

Rhizosphere soil genomic DNA extraction was carried out utilizing the PowerSoilfi DNA Isolation Kit (MoBio Laboratories, Carlsbad, CA, USA). The quality and concentration of the extracted soil genomic DNA were assessed by 1% (*w*/*v*) agarose gel electrophoresis and ultraviolet spectrophotometry. For library construction, sequencing libraries were prepared using the NEBNext Ultra DNA Library Prep Kit for Illumina (NEB, Ipswich, MA, USA) strictly in accordance with the manufacturer’s instructions. Each sample was uniquely barcoded by adding index codes to facilitate subsequent sequence assignment. Prior to PCR amplification and Illumina sequencing, the DNA was fragmented to an average size of 350 bp via sonication, followed by end-polishing, A-tailing, and ligation with full-length adaptors. Finally, metagenomic sequencing was conducted on the Illumina HiSeq PE150 platform, integrated with the Agilent2100 Bioanalyzer (Agilent Technologies, Santa Clara, CA, USA), following the protocol outlined in a previous study [48]. Each treatment contained 6 independent biological replicates for rhizosphere soil metagenomic sequencing.

### 4.5. Soil Bacterial Community Structure Analysis

After sequencing, raw reads were quality-filtered to remove low-quality sequences. Clean reads were assembled to generate contigs, and non-redundant gene catalogs were constructed for subsequent functional and taxonomic annotation. Taxonomic classification of bacterial genes was performed by alignment against the Genome Taxonomy Database (GTDB, https://gtdb.ecogenomic.org/, accessed on 1 December 2025) [49]. In the analysis of soil bacterial community structure, alpha diversity indices were employed to assess the richness and diversity of the microbial ecosystem. Specifically, the sobs index was utilized to quantify the number of operational taxonomic units (OTUs) present in the rhizosphere soils. The coverage index served as an indicator of sequencing depth, providing insights into the extent to which community richness was captured. The chao1 and ace indices were used to evaluate community abundance, while the Shannon and Simpson indices were applied to gauge community diversity. Additionally, beta diversity analysis was performed, encompassing PCoA and hierarchical tree-based clustering analysis, to statistically determine significant differences among diverse groups, following the methodology outlined in a previous study [50].

### 4.6. UHPLC-MS/MS-Based Soil Nontargeted Metabolomics

For UHPLC-MS/MS-based soil nontargeted metabolomics analysis, rhizosphere soil samples were dispatched to Majorbio (Shanghai, China) for processing in accordance with previously established protocols [19]. Briefly, approximately 50 mg of each soil sample was subjected to extraction by vortexing for 30 min in 400 µL of a methanol/water (4:1, [*v*/*v*]) solution. The resulting mixture was then allowed to settle at −20 °C before being treated with a high-throughput tissue crusher (Shanghai Wanbo Biotechnology Co., Ltd, Shanghai, China) at 50 Hz for 6 min, followed by an additional 30 s of vortexing and 30 min of ultrasonication at 40 kHz and 5 °C. After centrifugation at 13,000 *g* for 15 min at 4 °C, the supernatant was carefully transferred to sample vials for chromatographic separation using an ExionLCTM AD system (AB Sciex LLC, Framingham, MA, USA) equipped with an ACQUITY UPLC BEH C18 column (100 × 2.1 mm; 1.7 mm; Waters, Milford, MA, USA). The UPLC system was coupled to a quadrupole time-of-flight mass spectrometer (Triple TOFTM5600þ, AB Sciex LLC, Framingham, MA, USA) fitted with an electrospray ionization source operating in both positive and negative modes. To ensure data quality and system stability, an equal mixture of all samples was prepared as a QC sample, which underwent the same treatment and testing procedures as the analytical samples. Metabolite variables were analyzed using PLS-DA to provide an overview of the metabolic data and visualize general clustering patterns, trends, or outliers. Statistically significant groups were identified based on VIP values > 1.0 and *p* < 0.05. Differential metabolites between the two groups were summarized and mapped onto their respective biochemical pathways through metabolic enrichment and pathway analysis using the KEGG (http://www.genome.jp/kegg/, accessed on 16 December 2025). All data analyses were conducted using the Majorbio online analysis platform (https://www.majorbio.com/, accessed on 16 January 2026), following the provided online instructions. Each treatment contained 6 independent biological replicates for rhizosphere soil nontargeted metabolomics.

### 4.7. Measurement of Peanut Hormone Contents

Peanut root tissues from 3 biological replicates per treatment were sampled at the end of drought stress on 8 July 2024, under different treatments, and the contents of ABA, GA_3_, JA, IAA, zeatin, and SA were determined using reverse-phase liquid chromatography-tandem mass spectrometry (LC-MS/MS) with multiple reaction monitoring (MRM), as previously described [51].

### 4.8. Ultrastructural Assays

For SEM observations, peanut roots and pods were sampled at harvest in 2024 from 3 biological replicates per treatment [52]. Samples were immediately fixed in 4% glutaraldehyde solution prepared in 0.1 M phosphate-buffered saline (PBS; pH 6.8). After five sequential washes with PBS (5, 10, 15, 20, and 30 min), the specimens were dehydrated through a graded ethanol series, vacuum-dried, and sputter-coated with gold. SEM imaging was performed using a JSM-6360LV microscope (JEOL Ltd., Tokyo, Japan). For TEM analysis, small tissue fragments from roots and pods of three peanut plants per group were excised and fixed in Karnovsky’s fixative (a mixture of 4% paraformaldehyde and 5% glutaraldehyde in 50 mmol L^−1^ phosphate buffer [pH 7.2]) for 5 h. Following incubation in 50 mmol L^−1^ phosphate buffer (2 h), samples were post-fixed with 2% osmium tetroxide for 14 h. Dehydration was carried out using a graded acetone series (30–100%, 15 min per concentration), followed by embedding in Spurr’s resin. Ultrathin sections (100 nm thickness) were prepared using a diamond knife on an Ultracut E ultramicrotome (Leica, Wetzlar,Germany) and mounted on formvar-coated copper grids (1 mm × 2 mm). Sections were stained with 2% uranyl acetate (15 min) and lead citrate (5 min) before examination under a TEM microscope (H7500; Hitachi High-Tech, Tokyo, Japan) equipped with a CCD Megaview camera (Olympus Soft Imaging Solutions GmbH, Münster, Germany), following established protocols [53].

### 4.9. Peanut Pod Yield Measurement

Twelve representative plants per treatment were selected for morphological evaluation at harvest time. Samples were heated at 105 °C for 30 min to inactivate enzymes, then dried to a constant weight at 80 °C [45]. Pod yield was evaluated by air-drying all harvested pods and weighing them to calculate the 100-pod weight. Seeds were extracted by manually removing shells, and the 100-seed weight was recorded.

### 4.10. Statistical Analysis

Data are presented as mean values ± standard error of the mean (SE), with error bars in graphical representations indicating variability across replicates. Prior to parametric testing, all data were assessed for normality using the Shapiro–Wilk test and for homogeneity of variances using Levene‘s test. When these assumptions were satisfied, statistical comparisons between soil treatment groups were performed using Student’s *t*-test (* *p* < 0.05; ** *p* < 0.01) and one-way ANOVA (*p* < 0.05) with Statistical Product and Service Solutions Statistics software (IBM SPSS Statistics 25; IBM Corporation, Armonk, NY, USA) [54]. Following significant ANOVA results, LSD and Duncan’s post hoc tests served as multiple comparison corrections to control false positive errors in pairwise comparisons. For metabolomic differential analysis, false discovery rate (FDR) correction was further implemented to adjust *p*-values for multiple testing. Graphical visualizations were generated with GraphPad Prism (version 9.0), ensuring consistency in data presentation.

## 5. Conclusions

Salt and drought represent the predominant challenges confronting agricultural productivity in saline-alkali areas, imposing severe constraints on peanut growth and yields. In this study, we uncovered that the simultaneous exposure to salt and drought stress triggers more severe ultrastructural disruptions, cell wall degradation, and tissue deformations in peanuts, accompanied by a marked decrease in pod yield compared to single salt stress. Integrated multi-omics and physicochemical evidence further illustrated that this aggravated growth suppression may arise from several interlinked rhizosphere disruptions: the depletion of nitrogen-cycling and plant growth-promoting beneficial rhizobacteria such as Proteobacteria, Bacteroidetes, *Rhodanobacter*, *Bradyrhizobium*, and *Mycobacterium*, and the reduced accumulation of sugar metabolites. These stressors synergistically may degrade rhizosphere functionality, forming a negative feedback loop that restricts nutrient supply and plant stress tolerance. From an agricultural perspective, these findings deliver clear practical implications for saline-alkali peanut cultivation: timely supplementary irrigation at the flowering stage is essential to stabilize rhizosphere bacterial community and soil metabolites, thereby alleviating yield losses in coastal saline-alkali farmlands.

## Figures and Tables

**Table 1:** Effects of combined drought and salt stress or single salt stress on the morphology and pod yield of peanuts.

Year	Treatment	Main Stem Height	Lateral Branch Length	Pods Per Plant	100-Pod Weight (g)	100-Seed Weight (g)	Kernel Rate to Pod (%)	Pod Yield Per Plant (g)
2023	S	28.13 ± 1.18 a	28.84 ± 0.97 a	14.91 ± 0.76 a	77.14 ± 2.78 a	22.64 ± 1.46 a	58.07 ± 1.28 a	12.11 ± 1.51 a
	SD	22.90 ± 1.56 b	24.32 ± 1.39 b	11.45 ± 0.95 b	69.26 ± 2.38 b	18.39 ± 0.75 b	53.83 ± 0.44 b	9.72 ± 0.35 b
2024	S	34.13 ± 0.88 a	36.37 ± 1.56 a	18.43 ± 0.65 a	87.36 ± 0.89 a	27.86 ± 0.85 a	61.12 ± 1.78 a	14.59 ± 0.82 a
	SD	30.37 ± 1.81 b	31.36 ± 1.69 b	15.36 ± 1.58 b	84.59 ± 1.08 b	24.53 ± 0.90 b	54.26 ± 1.78 b	11.36 ± 0.64 b

Different lowercase letters indicate significant difference at *p* < 0.05 among the treatments.

**Table 2:** Alpha diversity indexes of different peanut rhizosphere soils.

Sample	Sobs	Ace	Chao	Shannon	Simpson	Shannoneven	Simpsoneven	Coverage
S1	4359	4359	4359	4.731538	0.042789	0.564623	0.005361	1
S2	4337	4337	4337	4.738587	0.04216	0.565806	0.005469	1
S3	4338	4338	4338	4.762588	0.04034	0.568656	0.005714	1
SD1	4352	4352	4352	4.713203	0.044917	0.562543	0.005116	1
SD2	4359	4359	4359	4.700252	0.046235	0.560889	0.004962	1
SD3	4361	4361	4361	4.763246	0.03993	0.568375	0.005743	1

**Table 3:** Differential metabolite-related metabolic pathway analysis.

Category	Pathway Description	DiffSet_Mix Metabolites (SD vs. S)	DiffSet_Mix Number
Carbohydrate metabolism	Galactose metabolism	Sucrose (down)	1
	Starch and sucrose metabolism	Sucrose (down)	
Amino acid metabolism	Glycine, serine and threonine metabolism	Ectoine (down)	2
	Lysine degradation	5-Aminopentanamide (up)	
Lipid metabolism	Linoleic acid metabolism	8(R)-Hydroperoxylinoleic acid (up)	1
Xenobiotics biodegradation and metabolism	Ethylbenzene degradation	Styrene (up)	1
	Styrene degradation	Styrene (up)	
Metabolism of cofactors and vitamins	Pantothenate and CoA biosynthesis	Dexpanthenol (up)	1
Biosynthesis of other secondary metabolites	Flavonoid biosynthesis	Pelargonidin (up)	2
	Anthocyanin biosynthesis	Pelargonidin (up)	
	Biosynthesis of various plant secondary metabolites	4-Hydroxycinnamoylagmatine (up)	
Chemical structure transformation maps	Biosynthesis of alkaloids derived from terpenoid and polyketide	Actinidine (up)	1
Biosynthesis of secondary metabolites	Biosynthesis of secondary metabolites	Actinidine (up)	4
		Pelargonidin (up)	
		4-Hydroxycinnamoylagmatine (up)	
		Sucrose (down)	
Signal transduction	Plant hormone signal transduction	N-Jasmonoylisoleucine (up)	1
Membrane transport	ABC transporters	Sucrose (down)	2
		Maltotriose (down)	
	Phosphotransferase system (PTS)	Sucrose (down)	

## Data Availability

All raw sequences for metagenomic sequencing are available in the China National Center for Bioinformation (https://www.cncb.ac.cn/, accessed on 19 November 2024) with accession number BioProject PRJCA032561 (https://ngdc.cncb.ac.cn/bioproject/browse/PRJCA032561, accessed on 19 November 2024), and all raw sequences for nontargeted metabolomics are available in the China National Center for Bioinformation with accession number PRJCA034910 (https://ngdc.cncb.ac.cn/bioproject/browse/PRJCA034910, accessed on 11 January 2025).

## Supplementary Materials

The following supporting information can be downloaded at: https://www.mdpi.com/article/10.3390/plants15142116/s1, Figure S1. Cladogram showing specific phylotypes of peanut rhizosphere soils responding to salt stress and combined stress. Indicator bacteria with linear discriminant analysis (LDA) scores of three or greater are highlighted, representing rhizobacterial communities associated with soils from single salt-treated and combined stress-treated groups. The circles denote phylogenetic levels ranging from phylum to genus (from the inner to the outer circle), with the diameter of each circle proportional to the abundance of the respective group. Figure S2. KEGG pathway analysis of differentially accumulated metabolites. Table S1. Total ion numbers and identification statistics.

## Abbreviations

The following abbreviations are used in this manuscript:

ROS	reactive oxygen species
ABA	abscisic acid
GA_3_	gibberellic acid
SA	salicylic acid
JA	jasmonic acid
PGPR	plant growth-promoting rhizosphere bacteria
S	single salt stress
SD	short-term drought exposure under salt stress at the flowering stage
SEM	scanning electron microscopy
TEM	transmission electron microscopy
PCoA	principal coordinate analysis
LDA	linear discriminant analysis
UHPLC-MS/MS	ultra-high-performance liquid chromatography-tandem mass spectrometry
KEGG	Kyoto Encyclopedia of Genes and Genomes
DAM	differentially accumulated metabolite
