# Supplementary material for: Salt–Drought Co-Stress Impairs Root Ultrastructure, Remodels Rhizosphere Bacteria, and Suppresses Peanut (Arachis hypogaea L.) Yield in Saline-Alkali Soil
Source: Plants (Basel). 2026 Jul 9;15(14):2116. doi: 10.3390/plants15142116 (PMC13414928; doi:10.3390/plants15142116)
Supplement: Supplementary file 1 [file plants-15-02116-s001.zip › plants-4399949-supplementary.pdf]

Figure S1

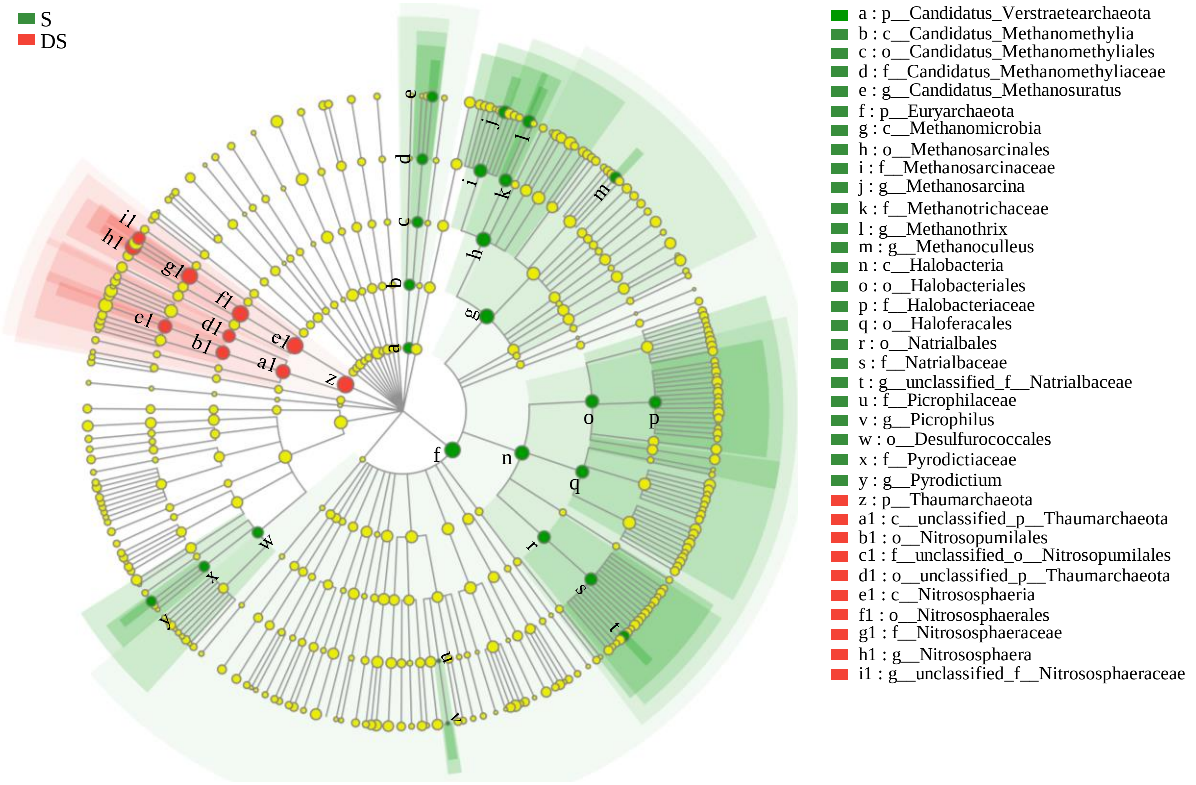

Figure S1. Cladogram showing specific phylotypes of peanut rhizosphere soils responding to salt stress and combined stress.
Indicator bacteria with linear discriminant analysis (LDA) scores of three or greater are highlighted, representing rhizobacterial communities associated with soil from single salt-treated and combined stress-treated groups. The circles denote phylogenetic levels ranging from phylum to genus (from the inner to the outer circle), with the diameter of each circle proportional to the abundance of the respective group.

Figure S2

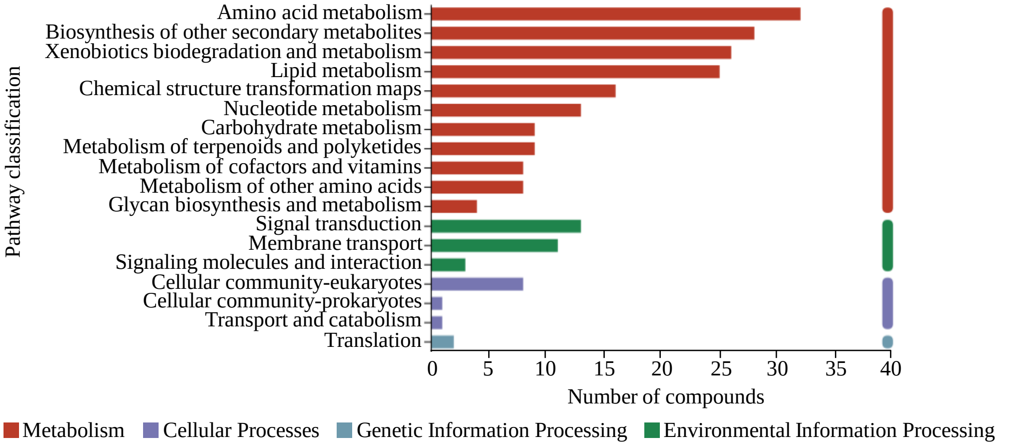

Figure S2. KEGG pathway analysis of differentially accumulated metabolites.

Table S1. Total ion numbers and identification statistics

Ion mode	All peaks	Identified metabolites	Metabolites in library	Metabolites in KEGG
pos	5797	645	547	239
neg	4063	176	140	44
